# Data on prevalence of atrial fibrillation and its association with stroke in low-, middle-, and high-income regions of China

**DOI:** 10.1016/j.dib.2018.06.082

**Published:** 2018-06-26

**Authors:** Xiaojun Wang, Qian Fu, Fujian Song, Wenzhen Li, Xiaoxv Yin, Wei Yue, Feng Yan, Hong Zhang, Hao Zhang, Zhenjie Teng, Longde Wang, Yanhong Gong, Zhihong Wang, Zuxun Lu

**Affiliations:** a Department of Social Medicine and Health Management, School of Public Health, Tongji Medical College, Huazhong University of Science and Technology, Wuhan, China; b School of Medicine and Health Management, Tongji Medical College, Huazhong University of Science and Technology, Wuhan, China; c Norwich Medical School, Faculty of Medicine and Health Science, University of East Anglia, Norwich, UK; d Department of Neurology, Tianjin Huanhu Hospital, Tianjin, China; e Department of Neurosurgery, Xuanwu Hospital, Capital Medical University, Beijing, China; f Department of Science and Education, People׳s Hospital of Deyang City, China; g Department of Neurology, Rizhao People׳s Hospital, Rizhao, China; h Department of Neurology, Hebei General Hospital, Shijiazhuang, China; i The National Health and Family Commission, Beijing, China; j Department of Neurosurgery, Shenzhen Second People׳s Hospital, Shenzhen University, Shenzhen, China

## Abstract

Data presented in this article are supplementary material to our research article entitled " Prevalence of Atrial Fibrillation in Different Socioeconomic Regions of China and Its Association with Stroke: Results from a National Stroke Screening Survey" (Wang et al., 2018) [1]. This data article summarizes previous studies of Atrial Fibrillation (AF) prevalence in China, and estimates the association between AF and stroke in different socioeconomic regions of China through a national survey.

**Specifications Table**

**Table:** 

Subject area	Epidemiology
More specific subject area	Cardiology
Type of data	SAS Data Set
How data was acquired	Standardized questionnaires, physical examinations, and blood samples
Data format	Raw and analyzed
Experimental factors	Socioeconomic regions were classified as low, middle, and high level according to the tertiles of per capita disposable income of households by regions in 2014
Experimental features	Stepwise logistic regression models were used to estimate the association between AF and stroke in different socioeconomic regions
Data source location	China Stroke Data Center, Stroke Control Project Committee Office of Nation Health and Family Planning Commission of PRC
Data accessibility	The data is with this article

**Value of the Data**

• These data will be of value for studies on comparing the epidemiological characteristics of AF in China.
• The data provides information on determinants of stroke in Low-, Middle-, and High-Income Regions of China.
• The data demonstrate that socioeconomic status should be taken into account by policymakers in relation to the prevention and control of AF related stroke.

## 1 Data

**Fig. 1:**
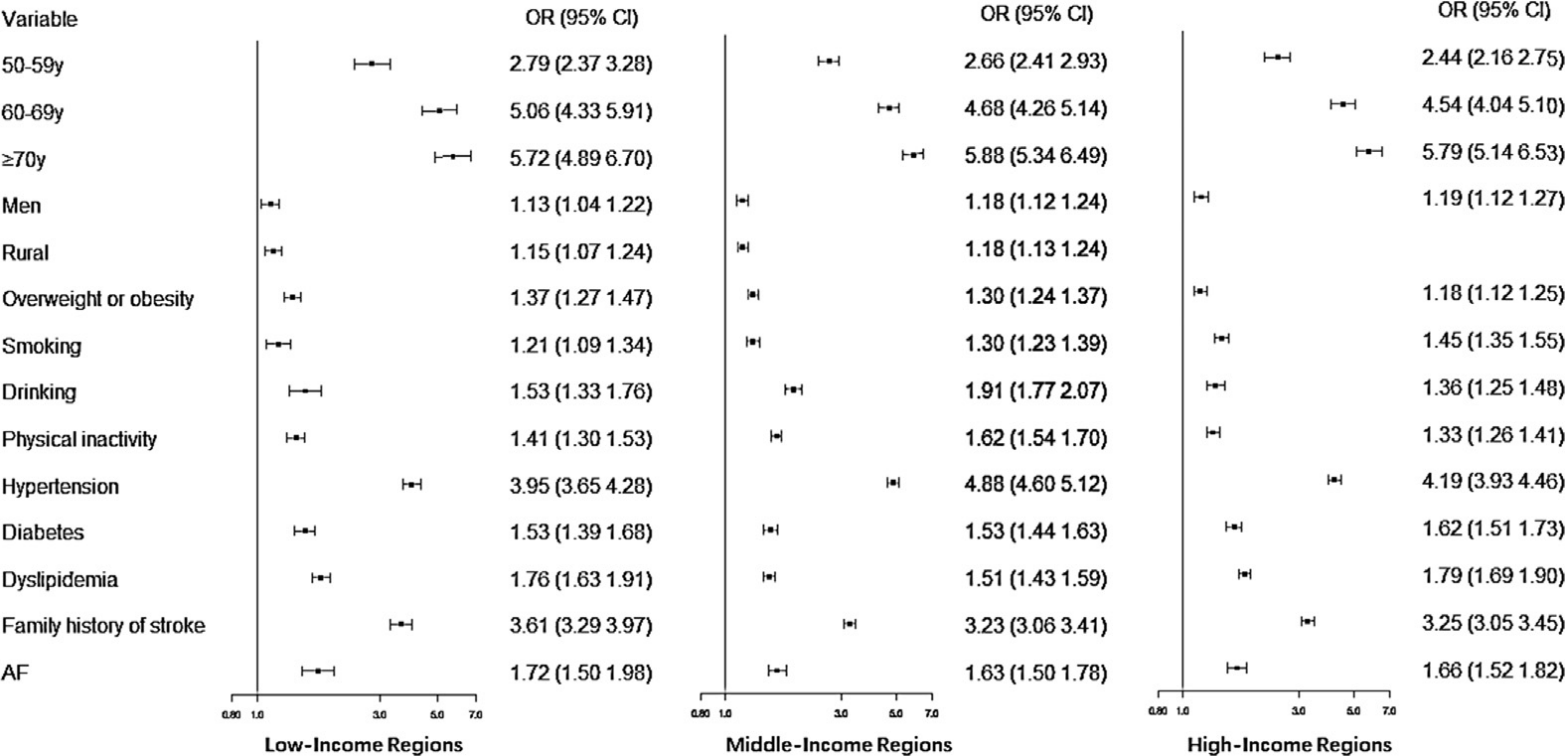
Association of risk factors with Stroke in Low-, Middle- and High-Income Regions. AF, Atrial Fibrillation. Adjust for age, Sex, location, overweight or obesity, smoking, drinking, physical inactivity, hypertension, diabetes, dyslipidemia, and a family history of stroke.

Fig. 1 shows the association between AF and stroke in low-, middle- and high-income regions. Table 1 summaries the representative data of AF prevalence in China.

**Table 1:** Summary of previous studies of AF prevalence in China.

Author, year	geographical regions	Study Population	*N*	Age	Study period	Diagnosis of AF	AF Prevalence					Stroke prevalence among patients with and without AF
							**Overall**	**Men**	**Women**	**Urban**	**Rural**	
Chan [2]	Hong Kong.	General	13,122	≥ 18 y	2014–2015	Smartphone-based wireless single-lead ECG and/or self-reported history	8.5%	10.6%	7.6%	–	–	AF vs non-AF: 10.0% vs 2.7%.
Li [3]	31 Chinese provinces	General	207,323	≥ 40 y	2013	ECG or self-reported history	1.57%	–	–	–	–	–
Han [4]	Jidong community in Hebei Province, northern China	General	8371	Mean age, 42.2±13.1 y	2013–2014	ECG or self-reported history	0.60%	0.76%	0.42%	–	–	–
Li [5]	9 provinces (Beijing, Sichuan, Shanxi, Heilongjiang, Jiangsu, Guangxi, Shaanxi, Guangdong, and Zhejiang.)	General	19,363	≥ 35 y	2004	Case history and ECG test.	Stand: 0.77% Crude: 1.03%	Stand: 0.78%	Stand: 0.76%	0.91%	0.67%	–
Lu [6]	Xinjiang province.	General	22,514	30–89 y	2009–2010	Medical history or ECG test	0.37%	0.5%	0.2%	–	–	AF vs non-AF: 7.2% vs 1.2%.
Zhang [7]	The China MUCA Study in 13 Populations, 10 of the 13 samples were included in the study.	General	18,615	≥ 35 y	2004	ECG test and history	1.04% (n=194)	–	–	–	–	–
Zhou [8]	13 provinces (Guangdong, Hebei, Henan, Hubei, Hunan, Inner Mongolia, Shandong, Shanxi, Sichuan, Tianjin, Yunan, Zhejiang, and Jiangxi).	General	29,079	30–85 y	2003	ECG test	Stand: 0.65%	Stand: 0.66%	Stand: 0.63%	–	–	AF vs non-AF: 12.95% vs 2.28%, OR = 2.776; 95% CI, 1.81- 4.25; *P* < 0.001.
							Crude: 0.77%					
Miao [9]	Xinjiang province.	Elderly	5398	≥ 60 y	2015	ECG or Holter recording.	Stand: 3.75% Crude: 3.56%	Crude: Uygur, 3.19%; Han, 5.01%	Crude: Uygur, 2.61%; Han, 3.31%	–	–	The prevalence of Ischemic stroke among AF and non-AF: Uygur: 8.82% vs 0.98%; Han: 6.08% vs 0.70%.
Li [10]	A newly urbanized suburban town in Shanghai province.	Elderly	3922	≥ 60 y	2006–2011	ECG test	1.8%	2.0%	1.6%	–	–	–
Chei [11]	CLHLS, 8 provinces (Shandong, Henan, Hubei, Hunan, Guangxi, Hainan Guangdong, and Jiangsu).	Elderly	1418	≥ 65 y	1998–2012	ECG test	3.5%	2.4%	4.5%	2.3%	4.6%	–
Sun [12]	Liaoning Province (including 26 rural villages).	Rural residents and most people are physical laborers engaged in heavy manual work.	11,956	≥ 35 y	2013	Medical history (diagnosed by a physician) and/or ECG test.	–	No significant Sex differences		–	1.2%.	–
Guo [13]	Yunnan Province, southwest of China	Urban residents.	471,446	≥ 20 y	2001–2012	ECG or Holter recording.	–	No significant Sex difference, but women aged > 70 years had a higher prevalence.		0.2%	–	AF vs non-AF: 6.4% vs 2.8%; OR = 2.28; 95% CI, 1.81–3.08; *P* < 0.001.
Yu [14]	Kailuan Coal Mining Corporation, North China.	Male employees and retired employees	81,061	18–98 y	2006–2007	ECG test	–	0.49%	–	–	–	–

AF, Atrial Fibrillation; ECG, electrocardiogram.

## 2 Experimental design, materials, and methods

The data of our study was from the China National Stroke Screening and Prevention Project (CNSSPP) in 31 provinces (except Tibet) in mainland China from October 2014 to November 2015. A total of 726,451 residents (386,975 women and 339,476 men) were included after the primary data cleaning. Socioeconomic regions were classified as low, middle, and high level according to the tertiles of per capita disposable income of households by regions in 2014 [14]. Data on demographic information, lifestyle risk factors, medical history, and a family history of stroke were collected through face-to-face interviews by a trained staff. We searched PUBMEN to identify population-based studies that reported prevalence of AF in China, and summarized findings in Table 1.

Stepwise logistic regression models were used to estimate the association between AF and stroke in different socioeconomic regions after adjusting for age, sex, location, overweight or obesity, smoking, drinking, physical inactivity, hypertension, diabetes, dyslipidemia, and a family history of stroke. Statistical analyses were performed by using SAS 9.3 for Windows (SAS Institute Inc., Cary, NC, USA), and in the two-tailed tests, a *P* value < 0.05 was considered statistically significant.
