# Supplementary material for: Data on prevalence of atrial fibrillation and its association with stroke in low-, middle-, and high-income regions of China
Source: Data Brief. 2018 Jun 26;19:1822–7. doi: 10.1016/j.dib.2018.06.082 (PMC6141785; doi:10.1016/j.dib.2018.06.082)
Supplement: Supplementary file 1 — Supplementary material [file mmc1.docx]

**Conflict of interest disclosures**

The authors report no relationships that could be construed as a conflict of interest.
